# Association between “cluster of differentiation 36 (CD36)” and adipose tissue lipolysis during exercise training: a systematic review

**DOI:** 10.3389/fphys.2023.1256440

**Published:** 2023-11-22

**Authors:** El Mokhtar El Ouali, Laurent Bosquet, Boutaina Elgharbaoui, Fatiha Laziri, Ismail Laher, Anthony C. Hackney, Azeddine Ibrahimi, Bouchra Taib, Sanae El Harane, Katja Weiss, Beat Knechtle, Abdelhalem Mesfioui, Hassane Zouhal

**Affiliations:** ^1^ Department of Biology, Laboratory of Biology and Health, Ibn Tofail University of Kenitra, Kenitra, Morocco; ^2^ University of Poitiers, Poitiers, France; ^3^ Biotechnology Lab (MedBiotech), Rabat Medical and Pharmacy School, Centre Mohammed VI for Research and Innovation (CM6), University Mohammed V, Rabat, Morocco; ^4^ Laboratoire Ecologie, Environnement et Santé Equipe Santé Humaine et Environnement Faculté des Sciences de Université Moulay Ismail, Meknès, Morocco; ^5^ Department of Anesthesiology, Pharmacology and Therapeutics, The University of British Columbia, Vancouver, BC, Canada; ^6^ Department of Exercise and Sport Science, University of North Carolina, Chapel Hill, NC, United States; ^7^ Institute of Sports Professions, Ibn Tofail University, Kenitra, Morocco; ^8^ Department of Pathology and Immunology, Faculty of Medicine, University of Geneva, Geneva, Switzerland; ^9^ University of Zurich, Zürich, Switzerland; ^10^ Movement, Sport, Health, and Sciences Laboratory (M2S) UFR-STAPS, University of Rennes 2-ENS Cachan, Rennes, France; ^11^ Institut International des Sciences du Sport (2IS), Irodouer, France

**Keywords:** fat oxidation, mitochondrial biogenesis, physical effort, muscle contraction, athletic performance

## Abstract

Fatty acid translocase (FAT/CD36) is a transmembrane glycoprotein belonging to the scavenger class B receptor family and is encoded by the cluster of differentiation 36 (CD36) gene. This receptor has a high affinity for fatty acids and is involved in lipid metabolism. An abundance of FAT/CD36 during exercise occurs in mitochondria and solitary muscles. As such, we aimed to systematically review the evidence for the relationship FAT/CD36 and adipose tissue lipolysis during exercise training. Five electronic databases were selected for literature searches until June 2022: PubMed, Web of Science, Scopus, science direct, and Google Scholar. We combined the different synonyms and used the operators (“AND”, “OR”, “NOT”): (CD36 gene) OR (CD36 polymorphism) OR (cluster of differentiation 36) OR (FAT/CD36) OR (fatty acid translocase) OR (platelet glycoprotein IV) OR (platelet glycoprotein IIIb) AND (adipose tissue lipolysis) OR (fatty acids) OR (metabolism lipid) OR (adipocytes) AND (physical effort) OR (endurance exercise) OR (high-intensity training). All published cross-sectional, cohort, case-control, and randomized clinical trials investigating CD36 polymorphisms and adipose tissue lipolysis during exercise in subjects (elite and sub-elite athletes, non-athletes, sedentary individuals and diabetics), and using valid methods to measure FAT/CD36 expression and other biomarkers, were considered for inclusion in this review. We initially identified 476 publications according to the inclusion and exclusion criteria, and included 21 studies investigating FAT/CD36 and adipose tissue lipolysis during exercise in our systematic review after examination of titles, abstracts, full texts, and quality assessments using the PEDro scale. There were nine studies with male-only participants, three with female-only participants, and nine studies included both female and male participants. There were 859 participants in the 21 selected studies. Studies were classified as either low quality (*n* = 3), medium quality (*n* = 13), and high quality (*n* = 5). In general, the data suggests an association between FAT/CD36 and adipose tissue lipolysis during exercise training. Improvements in FAT/CD36 were reported during or after exercise in 6 studies, while there were no changes reported in FAT/CD36 in 4 studies. An association between fat oxidation and FAT/CD36 expression during exercise was reported in 7 studies. No agreement was reached in 5 studies on FAT/CD36 content after dietary changes and physical interventions. One study reported that FAT/CD36 protein expression in muscle was higher in women than in men, another reported that training decreased FAT/CD36 protein in insulin-resistant participants, while another study reported no differences in FAT/CD36 in young, trained individuals with type 2 diabetes. Our analysis shows an association between FAT/CD36 expression and exercise. Furthermore, an association between whole-body peak fat oxidation and FAT/CD36 expression during exercise training was demonstrated.

Systematic Review Registration: [PROSPERO], identifier [CRD42022342455]

## 1 Introduction

Lipolysis of adipose tissue and fat oxidation plays a crucial role in physical training, whereby the fat stored in the adipose tissue is converted to fatty acids that can be used for energy production during exercise. Intense and prolonged training and insufficient carbohydrate intake can limit muscle glycogen stores and reduce physical performance (Achten et al., 2004; Rodriguez et al., 2009; de Sousa et al., 2010). Intramyocellular lipids are the most important substrates for adenosine triphosphate (ATP) production in skeletal muscle during aerobic exercise (van Loon, 2004). During low to moderate intensity exercise, long-chain fatty acids (LCFAs) are the main source of energy for skeletal muscle, particularly for type 1 fibers (Muscella et al., 2020). Some studies suggest that cellular uptake of LCFAs may be increased during dietary interventions and during exercise training (Kiens et al., 2004).

At lower exercise intensities, there is an increase in fat oxidation, which peaks at a workload corresponding to maximal fat oxidation (MFO). However, as the exercise intensity load increases beyond this point, fatty acid oxidation decreases and the main source of ATP production switches to carbohydrate (CHO) oxidation (Brooks and Mercier, 1994). Venables et al. (Venables et al., 2005) found in their study of 300 men and women performing progressive treadmill exercise that MFO occurred at a workload of 48% of peak oxygen consumption (VO_2_ peak) and 61% of maximal heart rate (HR_max_). After 3 months of continuous moderate-intensity training (MICT), there was an increase in whole-body fat oxidation and a 41% decrease in muscle glycogen utilization during prolonged exercise (Hurley et al., 1986). Skeletal muscle fat oxidation is influenced by many factors, including capillary density, type 1 (I) fibers percentage, mitochondrial protein expression rate, and enzyme activities, as well as enzymes involved in β-oxidation and hydrolysis of intramuscular triacylglycerols (Nordby et al., 2006; Stisen et al., 2006; Dandanell et al., 2018; Shaw et al., 2020). Individuals with obesity tend to have lower mRNA levels in skeletal muscle for genes involved in beta-oxidation and mitochondrial biogenesis, compared to subjects without obesity (Baker et al., 2015). In patients with type 2 diabetes, adipokine profiles can be improved by long-term aerobic and resistance training combined with dietary modifications (Zouhal et al., 2021). Longer high-intensity intermittent training (HIIT) training durations can lead to greater increases in fat oxidation, as shown in young inactive women who underwent 12 weeks of HIIT who experienced a 16%–26% increase in fat oxidation and a 20% increase in MFO (Astorino et al., 2013).

Exercise training upregulates the mRNA of proteins involved in substrate metabolism in human skeletal muscle, including increases in proteins involved in oxidative metabolism (Pilegaard et al., 2000). Several proteins play crucial roles in facilitating the transport of fatty acids from adipose tissue to muscle mitochondria and their subsequent metabolism (Holloway et al., 2009). The CD36 gene encodes a transmembrane glycoprotein known as Cluster of Differentiation 36 (CD36), which also has various names such as fatty acid translocase (FAT), FAT/CD36, platelet glycoprotein IV, and IIIb (Gong et al., 2017). This glycoprotein is classified in the class B scavenger receptor family (Gong et al., 2017), and is found on the surface of adipocytes, hepatocytes, macrophages, skeletal and cardiac myocytes, as well as intestinal epithelial cells, kidney, breast, platelets, and microvascular endothelial cells (Febbraio et al., 2001). This receptor has a high affinity for fatty acids, and is involved in lipid metabolism (Gilbertson and Khan, 2014). Additionally, FAT/CD36 serves as the primary protein responsible for transporting LCFAs in both sarcolemmal and mitochondrial membranes (Bezaire et al., 2006; Glatz et al., 2022). Specifically, it has been shown that FAT/CD36 is located on the outer mitochondrial membrane, preceding the action of long-chain acyl-CoA synthetase in fatty acid activation (Smith et al., 2011), as recently confirmed by Zeng et al. (2022). Thus, levels of FAT/CD36 increase in mitochondria and solitary muscle during endurance exercise (Talanian et al., 2010).

The CD36 gene is located on chromosome 7 (q11.2) contains 15 exons and covers approximately 32 Kb (Yun et al., 2007). The CD36 gene contains a single nucleotide polymorphism (SNP), in particular the variation rs1761667 which is a G > A substitution, located in the 5′ intron flanking exon 1A (Daoudi et al., 2015). Clinical studies report an association of this polymorphism with cardiovascular disease (Zhang et al., 2014; Boghdady et al., 2016), type 2 diabetes mellitus (T2DM), and obesity (Banerje et al., 2010). A study of 722 European individuals with obesity with the SNP-78A > C FAT/CD36 variant (rs2232169) indicates no significant difference in fat oxidation under fasting conditions, but increases occurred after consumption of a high-fat diet (Corpeleijn et al., 2010). According to Bokor et al. (2010) four SNPs in the CD36 gene (rs3211908, rs3211867, rs3211883, and rs1527483) have been associated with an increased risk of obesity in adolescents.

Several previous findings confirm that physical activity stimulates both lipolysis of adipose tissue and oxidation of fatty acids (Hurley et al., 1986; Brooks and Mercier, 1994; Venables et al., 2005; Stisen et al., 2006; Shaw et al., 2020). However, fatty acid oxidation is under the control of numerous biomarkers and proteins, including FAT/CD36 and various factors associated with mitochondrial biogenesis (Pilegaard et al., 2000; Holloway et al., 2009; Talanian et al., 2010; Astorino et al., 2013; Gilbertson and Khan, 2014). Accordingly, the impact of physical training on FAT/CD36 content during and after exercise is unclear (Glatz et al., 2022). To the best of our knowledge, no systematic review has yet explored the relationship between FAT/CD36 and adipose tissue lipolysis during exercise training. Consequently, we aim to achieve this objective by conducting a systematic review of previous studies examining the relationship between FAT/CD36 and adipose tissue lipolysis in response to exercise training.

## 2 Methods

The current protocol was registered in PROSPERO (registration number CRD42022342455). This systematic review was carried out according to the recommendations of the Cochrane Handbook for the Systematic Review and Meta-Analysis of Interventions (Furlan et al., 2009). A bibliographic search strategy was carried out in accordance with the Preferred Reporting Items for Systematic Reviews and Meta-Analyses (PRISMA) guidelines (Moher et al., 2009).

### 2.1 Search strategy and inclusion/exclusion criteria

Five electronic databases were searched for publications until June 2022: PubMed, Web of Science, Scopus, Science Direct, and Google Scholar. We used the PICOS framework, which considers Population, Intervention, Comparison, Outcome and Study design as key criteria (Moher et al., 2015), as summarized in Table 1. However, our inclusion criteria were published (i) cross-sectional, cohort, case-control, and randomized clinical trials, (ii) studies investigating FAT/CD36 and adipose tissue lipolysis during exercise in subjects (elite and sub-elite athletes, non-athlete, sedentary and diabetic), and (iii) studies using valid methods to measure the expression of FAT/CD36 and other biomarkers. Studies were excluded from the systematic review if they: (i) were review articles (ii) were carried out in pregnant or breastfeeding women, (iii) involved children, or adolescents aged < 16 years. To avoid the risk of missing studies, we combined the different synonyms and used the operators (“AND”, “OR”, “NOT”): (CD36 gene) OR (CD36 polymorphism) OR (cluster of differentiation 36) OR (FAT/CD36) OR (fatty acid translocase) OR (platelet glycoprotein IV) OR (platelet glycoprotein IIIb) AND (adipose tissue lipolysis) OR (fatty acids) OR (metabolism lipid) OR (adipocytes) AND (physical effort) OR (endurance exercise) OR (high-intensity training). Additionally, the references of the reviewed articles allowed us to identify other relevant studies.

**TABLE 1:** PICOS (participants, interventions, comparisons, outcomes, study design). Adapted from (Moher et al., 2015), licensed CC-BY-4.0.

PICOS element	Details
Participants	Elite and sub-elite athletes, non-athlete, sedentary and diabetic subjects
Interventions	Measurement of CD36 expression, before and/or after monitoring physical effort alone or with dietary intervention
Comparisons	Before/after physical effort and dietary intervention
Outcomes	Increased or decreased CD36 expression
Study designs	Case-controls, cohorts and cross sections, nRCTs, nRnCTs, RCTs

nRCT, non-randomized controlled trial; nRnCT, non-randomized non-controlled trial; RCT, randomized controlled trial.

### 2.2 Study selection process

The screening and selection of studies were performed by 2 authors (E.E and B.E) independently and were based on the inclusion/exclusion criteria described above. When the article title and abstract suggested an association with FAT/CD36, adipose tissue lipolysis, and physical effort, the full text was reviewed. Any disagreement on one or more studies was discussed with all reviewers until a consensus was reached.

### 2.3 Data extraction

For this systematic review, the inclusion criteria were based on the PICOS (Population, Intervention, Comparison, Outcome, and Study Design) framework (Moher et al., 2015). For each eligible study, these data are extracted: author and year of publication, country, number and sex of participants, anthropometric data, notes about participants, the purpose, intervention, and outcome of each study. For missing data or additional details, study authors were contacted by email.

### 2.4 Quality assessment

Assessment of the methodological quality of eligible studies was carried out by two authors independently (E.E and S.E). To assess the methodological quality of each study, we used the Physiotherapy Evidence Database (PEDro) scale (Moher et al., 2009), whose reliability and validity have been demonstrated (Maher et al., 2003). The PEDro scale assesses studies in terms of external validity (criterion 1), internal validity (criteria 2–9), and adequacy of statistical information to interpret the results (criteria 10–11). This assessment tool uses a scoring system ranging from 0 to 11 points, with higher scores indicating better methodological quality. A score of 1 indicates that the study met the assessment criteria and a score of 0 indicates dissatisfaction. Due to the inherent characteristics of physical activity interventions, it could be difficult to implement patient and therapist blinding and appropriate allocation methods. Therefore, the maximum score that a trial could achieve on the scale was 8 points. Each study with a threshold of 6 or more on the PEDro scale was considered a high-quality study (Maher et al., 2003). When there was a disagreement on a study between the two reviewers, the other authors were consulted for a supplementary assessment.

### 2.5 Levels of evidence

Levels of evidence are determined according to the methodological quality and statistical heterogeneity of the studies (van Tulder et al., 2003; Neal et al., 2016). Strong evidence requires pooled results from at least three studies, including two high-quality, statistically homogeneous studies. Moderate evidence comes from several statistically heterogeneous studies, including one good-quality study or several statistically homogeneous studies of moderate/low quality. Limited evidence was obtained from a single high-quality study or several statistically heterogeneous studies of moderate/low quality. Very limited evidence was obtained from a single study of moderate or low quality. No evidence was found when the pooled results were not statistically significant and came from several statistically heterogeneous studies.

## 3 Results

### 3.1 Selection of studies and characteristics of included studies

**FIGURE 1:**
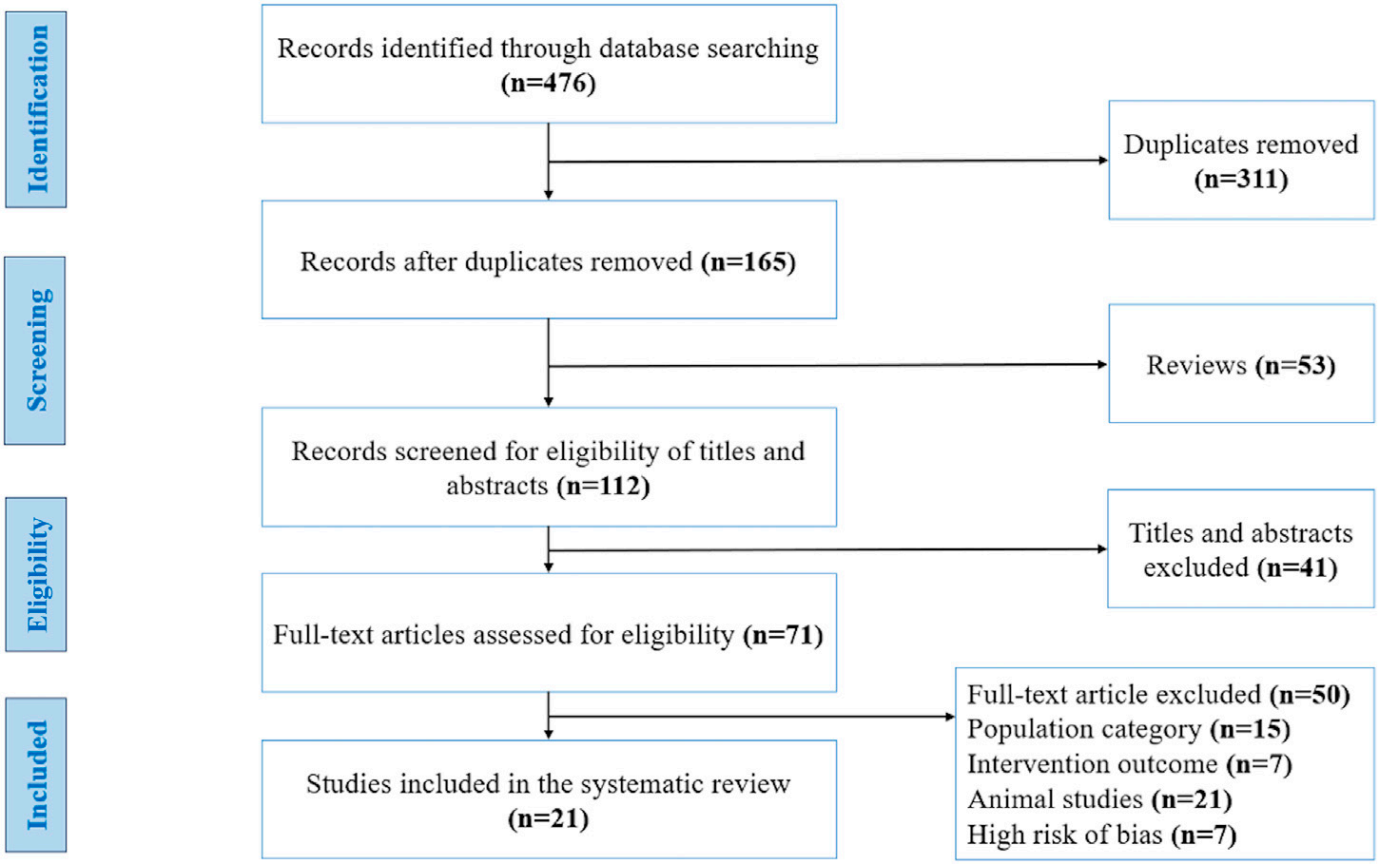
Flow diagram of studies included in this systematic review, using the recommendations in the Preferred Reporting Items for Systematic Reviews and Meta-Analyses (PRISMA) statement. Adapted from (Moher et al., 2009), licensed under Creative Commons Attribution License.

We identified 476 publications at the start of our literature search, of which 311 studies were duplicates, 53 were reviews and 41 articles were ineligible due to their title and abstract. The full texts of 71 articles were carefully evaluated and resulted in the exclusion of 50 articles for the following reasons: population category (*n* = 15), intervention outcome (*n* = 7), animal studies (*n* = 21), and high-risk of bias (*n* = 7). Finally, based on the inclusion and exclusion criteria and quality assessment, 21 studies were included in our systematic review (Figure 1). These studies were carried out in 9 different countries: Australia, Canada, United States of America, United Kingdom, Finland, Denmark, Taiwan, New Zealand, and Japan. The studies have a collective sample size of (*n* = 859 participants), of which 9 studies involved only men, 3 studies involved only women, and 9 studies involved both women and men. The general characteristics of the 21 studies included in the systematic review are summarized in Table 2.

**TABLE 2:** Characteristics of studies included in the systematic review on the association between cluster of differentiation 36 (CD36) and adipose tissue lipolysis during physical effort.

Studies (Author, year).	Country	Sample size and sex	BMI (kg/m^2^)/BM (kg)/(mean ± SD/SE or range)	Mean age (years)/(mean ± SD/SE or range)	Participants	Purpose of the study	Intervention	Outcome
Arkinstall et al. (2004)	Australia	7 men	80.3 ± 9.5	33 ± 5	Subjects moderately trained in cycling	To quantify the acute effect of L-CHO and H-CHO on energy metabolism genes transcription	L-CHO (0.7 g/kg of BM of CHO, 4.4 g/kg BM of fat, 4 g/kg BM of protein) or H-CHO (10 g/kg BM of CHO, 1 g/kg BM of fat, 1.9 g/kg BM of protein)	FAT/CD36 increased following an acute L-CHO and short-term exercise
Bruce et al. (2003)	Australia	27 men and women	TYPE 2: 29.1 ± 1.8, OLD: 27.6 ± 1.0, YOUNG: 26.2 ± 1.8, TRAINED: 23.2 ± 0.3 (kg/m^2^)	TYPE 2: 46 ± 3, OLD: 50 ± 2, YOUNG: 25 ± 2, TRAINED: 25 ± 1	8 patients with type 2 diabetes, 10 healthy but sedentary and 9 well-trained endurance athletes	To determine whole-body insulin sensitivity, LCACoA content, TGm concentration, fatty acid transporter protein content, and oxidative enzyme activity	determination of VO2 peak. Muscle biopsies from the vastus lateralis were taken before and after a 2-h euglycemic-hyperinsulinemia clamp	No differences in CD36 protein content between the TYPE 2, YOUNG, and TRAINED groups
Burgomaster et al. (2007)	Canada	16 men	EG: 80 ± 4, CG: 76 ± 3 (kg)	22 ± 1 (EG) and 26 ± 1 (CG)	Subjects were active students	To examine the early time course for changes in metabolite transport proteins in human skeletal muscle in response to HIIT	4–6 × 30 s all-out cycling efforts with 4-min recovery, 3 days/wk for 6 wk are performed	CD36 remained unchanged during training and detraining compared to baseline data
Civitarese et al. (2005)	United States of America	7 men	23.8 ± 1.0 (kg/m^2^)	22.7 ± 0.6	Healthy untrained men	the effects of a single 2-h bout of moderate-intensity exercise on the expression of key genes involved in fat and carbohydrate metabolism with or without glucose ingestion	50% of maximum power is exerted, then ingestion of 1.4 and 0.35 g/kg body wt glucose solution	Glucose ingestion during moderate-intensity exercise inhibits CD36 expression
Frandsen et al. (2022)	Denmark	14 men	Young (n = 7): 23.6 ± 0.8, Old (n = 7): 23.4 ± 1.2 (kg/m^2^)	Young (n = 7): 30 ± 5.0, Old (n = 7): 65 ± 6	Older and younger men	The physiological and metabolic impact of prolonged repeated exercise of moderate intensity (7–10 h/day for 15 consecutive days at ≈63% HRmax)	Participants travelled ≈3000 km by bicycle over 15 days	CD36 unchanged after 15 days of extreme endurance exercise
Fujii et al. (2019)	Japan	495 men and women	GG (n = 268): 62·9 ± 9.7, GA (n = 190): 64 ± 10.1, AA (n = 37): 64.9 ± 9.9 (kg)	GG (n 268): 23.6 ± 3.4, GA (n 190): 23.7 ± 3.4, AA (n 37): 23.4 ± 2.8	Individuals participated in a health check-up carried out in Hokkaido, Japan	To investigate the associations of two CD36 polymorphisms (rs1761667 and rs1527483) and dietary fat intake and metabolic phenotypes	Dietary nutrient intake was estimated and mean blood pressure (SBP and DBP) was calculated	The AA genotype of rs1761667 in the CD36 gene was associated with higher intake of total fat
Hammond et al. (2016)	United Kingdom	10 men	75.9 ± 6 (kg)	24 ± 1.5	Trained male runners	To examine the effects of reduced CHO but high post-exercise fat availability on cell signaling and expression of genes with putative roles in regulation of mitochondrial biogenesis, lipid metabolism and muscle protein synthesis	A twice per day exercise model (3.5 h between sessions) comprising morning HIIT (8 x 5-min at 85% VO_2_ peak) and afternoon MICT (60 min at 70% VO_2_ peak)	CD36 is higher in HFAT during recovery period after MICT exercise compared to HCHO
Hearris et al. (2019)	United Kingdom	8 men	76.0 ± 12.7 (kg)	22 ± 3	Recreationally active men	To examine the effects of graded pre-exercise glycogen concentrations on both exercise capacity and the modulation of selected skeletal muscle signaling pathways with putative roles in the regulation of mitochondrial biogenesis	Glycogen-depleting cycling exercise followed by the consumption: L-CHO: 0, M-CHO: 3.6 and H-CHO: 7.6 g/kg across a 6-h period	Neither exercise nor CHO availability affected the mRNA expression of CD36
Holloway et al. (2006)	Canada	15 men and women	24 ± 1 (kg/m^2^)	22 ± 1	Recreationally active individuals	The effects of exercise on CPTI palmitoyl-CoA and M-CoA kinetics and FAT/CD36 on skeletal muscle mitochondria	120 min of cycling at ∼60% VO_2_ peak	CD36 protein increased during exercise
Honkala et al. (2020)	Finland	54 men and women	Healthy men: 26.1 ± 2.4, IR men: 30.4 ± 2.9, IR women: 30.5 ± 2.5 (kg)	Healthy men: 49 ± 8, IR men: 47 ± 3, IR women: 54 ± 6	Middle-aged, physically inactive, healthy men and insulin resistance men and women	The impact of HIIT and MICT on adipocyte morphology and adipose tissue metabolism and function	2-weeks of HIIT (4–6×30s at maximum effort) and MICT (40–60 min at 60% of VO_2_ peak)	In IR participants, training increased adipose tissue vasculature and decreased CD36
Jayewardene et al. (2016)	Australia	34 men and women	“rs1527479”: C (24): 23.4 ± 2.7; TT (10): 23.9 ± 2.1, “rs1984112”: G (21): 23.5(9.3); AA (13): 23.2(7.9) (kg/m^2^)	“rs1527479”: C (24): 22.6 ± 3.6; TT (10): 22.7 ± 3.4, “rs1984112”: G (21): 22.0 ± 3.2; AA (13): 23.8 ± 3.7	Physically active participants	Two SNPs (rs1527479 and rs1984112) were assessed for associations with whole-body substrate oxidation	Response to a 75g (OGTT), fasting plasma lipids and CVD risk factors, following a 4-weeks endurance exercise training program.	TT SNP carriers at rs1527479 and GG carriers at rs1984112 were associated significantly with greater whole-body rate of fat oxidation
Juan et al. (2021)	Taiwan	26 men and women	23.0 ± 2.2 (kg/m^2^)	46.9 ± 9.0	Ultra-marathon runners’ men and women	To measure the expression of ABC transporters and scavenger receptor mRNAs	100 km ultramarathon event	CD36 is increased in PBMCS after endurance exercise
Kiens et al. (2004)	Denmark	46 men and women	F (NT = 64.9 ± 2.8, MT = 59.8 ± 2.0, ET = 64.4 ± 2.7) and M (NT = 82.9 ± 5.7, MT = 76.4 ± 1.7, ET = 75.2 ± 1.8) (kg)	F (NT = 27 ± 1, MT = 27 ± 1, ET = 25 ± 1) and M (NT = 27 ± 2, MT = 23 ± 1, ET = 26 ± 1)	Nontrained, moderately trained and endurance trained	Assess whether physical activity and sex influence lipid metabolism and mRNA transcript levels of several lipid-binding proteins	Bicycle ergometer at 60% peak VO_2_ for 90 min	CD36 protein in muscle was higher in women than in men
Maunder et al. (2022)	New Zealand	17 men	80.5 ± 9.6 (kg)	34 ± 7	Endurance-trained male cyclists and triathletes	To assess the relationships between PFO measured during fasting, CD36 abundance, endurance performance, and fat oxidation rates during MICT	Incremental cycling exercise test to evaluate the PFO	CD36 is correlated with PFO during exercise
Perry et al. (2008)	Canada	8 men and women	72.7 ± 4 (kg)	24 ± 1	Participate in some form of aerobic activity (cycling and jogging)	To investigate the ability of 6 wk of HIIT (18 h at 90% of VO_2_ peak) to increase the whole-body CHO and Fat oxidation	6 weeks of cycle-ergometer HIIT: ∼1 h of 10 × 4 min intervals at ∼90% of VO_2_ peak, separated by 2 min rest, 3 d/wk	6 weeks of HIIT (3 d/week) increases fat oxidation and CD36
Riis et al. (2019)	Denmark	18 men	LOW‐CHO (n = 6) 22.0 ± 1.6, HIGH‐CHO (n = 7) 23.0 ± 1.5 (kg/m^2^)	LOW‐CHO (n = 6) 28 (23–37), HIGH‐CHO (n = 7) 27 (24–44)	Endurance‐trained male	To investigate the sustained effects on MFO rate and proteins involved in intramuscular lipid metabolism	L-CHO and H-CHO groups, follow 3 training blocks/wk for 4 weeks, with HIIT and MICT	No difference of CD36 in L-CHO compared to H-CHO during exercise
Schenk and Horowitz (2006)	United States of America	15 women	n = 7 for DIET (34.9 ± 1.8) and n = 8 for EX + DIET (31.7 ± 0.9) (kg/m^2^)	n = 7 for DIET (30 ± 3) and n = 8 for EX + DIET (30 ± 2)	Women with abdominal obesity	To evaluate the effects of adding endurance exercise training to a weight loss program on the localization of FAT/CD36 in skeletal muscle from humans with obesity and whether such changes are associated with changes in fat oxidation	Caloric intake of 500–800 kcal/day less than that required to maintain body weight. 45 min 3 d/wk of stationary cycling at 70%–85% of HR_max_	Physical training alters the localization of CD36 and increases its association with CPT I, and increase fat oxidation
Talanian et al. (2007)	Canada	8 women	65.0 ± 2.2 (kg)	22 ± 1	Healthy recreationally active women	To examine the effects of HIIT sessions over 2 wk on skeletal muscle fuel content, mitochondrial enzyme activities, fatty acid transport proteins, VO_2_ peak and whole body metabolic, hormonal, and cardiovascular responses to exercise.	7 supervised HIIT sessions in 13 days: 10 cycling sessions of 4 min at 90% of peak VO2 separated by 2 min of rest.	CD36 was not affected after 2 weeks of HIIT
Talanian et al. (2010)	Canada	10 women	65 ± 2 (kg)	22 ± 1	Healthy females untrained but engaged in light recreational physical activity 2 days/wk.	To determine whether HIIT increases total skeletal muscle, sarcolemmal, and mitochondrial membrane fatty acid transport protein contents.	6 weeks of HIIT with 3 days/week: ten 4-min cycling bouts at 90% VO_2_ peak separated by 2 min of rest.	6 weeks of HIIT does not change CD36
Thomas et al. (2012)	United Kingdom	17 men	G1 (n = 9): 80 ± 11, G2 (n = 8): 67 ± 6 (kg)	G1 (n = 9): 32 ± 8, G2 (n = 8): 27.8 ± 6.4	Healthy active untrained individuals	Testing whether exercise is associated with the generation of ligands (PPAR γ) in plasma	Compare the response to an intense exercise session (24 h follow-up) and an 8-week training program comprising multiple exercise sessions	CD36 expression was significantly increased exercise program
Tunstall et al. (2002)	Australia	7 men and women	22.6 kg/m^2^ (range 17–26 kg/m^2^)	28.9 ± 3.1	Healthy, untrained subjects	The effects of a single session of exercise and physical training on the expression of genes necessary for the transport and oxidation of FA, as well as on the gene expression of transcription factors involved in the regulation of FA homeostasis have been studied.	9 days of 60 min cycling per day at 63% VO2 peak	9 consecutive days of aerobic training increased total lipid oxidation and CD36 expression

LCACoA; long-chain fatty acyl coenzyme A, TGm; skeletal muscle triglyceride, SNPs; single nucleotide polymorphisms, OGTT; oral glucose tolerance test, CVD; cardiovascular disease, PBMCS; peripheral blood mononuclear cells, MFO; maximal fat oxidation, PFO; peak whole-body fat oxidation, PPAR; peroxisome proliferator-activated receptor- γ, [GG], [GA], [AA], [TT], genotype; low carbohydrate (L-CHO), medium carbohydrate (M-CHO), high carbohydrate (H-CHO); group A (GA), group B (GB), group 1, 2 (G1, G2); Untrained Female/Male (NTF/M), Moderately Trained Female/Male (MTF/M), Endurance Trained Female/Male (ETF/M); continuous exercise (CON), high intensity intermittent training (HIIT). data is within (mean ± SD/SE or range).

### 3.2 Study quality assessment

The quality of the studies selected was generally satisfactory according to the Physiotherapy Evidence Database (PEDro) scale (Moher et al., 2009). We identified 3 low-quality studies (less than 4 points), 13 studies of medium quality (more than 4 and less than 6 points), and 5 studies of high quality (more than 6 points), as shown in Table 3.

**TABLE 3:** Physical Therapy Evidence Database (PEDro) score of included studies.

Study	Assessment criteria											PEDro score	Quality
	1	2	3	4	5	6	7	8	9	10	11		
Arkinstall et al. (2004)	1	1	0	1	0	0	0	0	1	1	1	6	High
Bruce et al. (2003)	1	0	0	1	0	0	0	0	1	0	1	4	Medium
Burgomaster et al. (2007)	1	0	0	1	0	0	0	0	1	1	1	5	Medium
Civitarese et al. (2005)	1	1	1	1	1	0	0	0	1	0	1	7	High
Frandsen et al. (2022)	1	0	0	0	0	0	0	0	1	0	1	3	Low
Fujii et al. (2019)	1	0	0	1	0	0	0	0	1	1	1	5	Medium
Hammond et al. (2016)	1	0	0	0	0	0	0	0	1	0	1	3	Low
Hearris et al. (2019)	1	0	0	1	0	0	0	0	1	1	1	5	Medium
Holloway et al. (2006)	1	1	0	1	0	0	0	0	1	1	1	6	High
Honkala et al. (2020)	1	1	1	1	0	0	0	0	1	0	1	6	High
Jayewardene et al. (2016)	1	0	0	1	0	0	0	0	1	1	1	5	Medium
Juan et al. (2021)	1	0	0	1	0	0	0	0	1	1	1	5	Medium
Kiens et al. (2004)	1	1	0	1	0	0	0	0	1	0	1	5	Medium
Maunder et al. (2022)	1	0	0	1	0	0	0	0	1	1	1	5	Medium
Perry et al. (2008)	1	0	0	1	0	0	0	0	1	1	1	5	Medium
Riis et al. (2019)	1	1	0	1	0	0	0	0	1	1	1	6	High
Schenk and Horowitz (2006)	1	0	0	1	0	0	0	0	1	1	1	5	Medium
Talanian et al. (2007)	1	0	0	0	0	0	0	0	1	1	1	4	Medium
Talanian et al. (2010)	1	0	0	0	0	0	0	0	1	1	1	4	Medium
Thomas et al. (2012)	1	0	0	1	0	0	0	0	1	1	1	5	Medium
Tunstall et al. (2002)	1	0	0	0	0	0	0	0	1	0	1	3	Low

### 3.3 Association between CD36 and adipose tissue lipolysis during exercise training

Our study assessed the association between FAT/CD36 and adipose tissue lipolysis during training. However, the results of the studies included in our systematic review assessed FAT/CD36 expression with different interventions: (i) after exercise (10 studies) (Tunstall et al., 2002; Holloway et al., 2006; Schenk and Horowitz, 2006; Burgomaster et al., 2007; Talanian et al., 2007; Perry et al., 2008; Talanian et al., 2010; Thomas et al., 2012; Juan et al., 2021; Frandsen et al., 2022), (ii) after diet and physical intervention (5 studies) (Arkinstall et al., 2004; Civitarese et al., 2005; Hammond et al., 2016; Hearris et al., 2019; Riis et al., 2019), (iii) in association with fat oxidation during exercise (7 studies) (Tunstall et al., 2002; Schenk and Horowitz, 2006; Perry et al., 2008; Jayewardene et al., 2016; Fujii et al., 2019; Honkala et al., 2020; Maunder et al., 2022), (iv) in the relation between individuals of different groups (type 2 diabetes, young people and trained subjects) (1 study) (Bruce et al., 2003), and (v) comparison of sex (1 study) (Kiens et al., 2004).

Regarding the relationship between FAT/CD36 expression and exercise, 6 studies (Tunstall et al., 2002; Holloway et al., 2006; Schenk and Horowitz, 2006; Perry et al., 2008; Thomas et al., 2012; Juan et al., 2021), reported an improvement during or after exercise, while 4 studies (Burgomaster et al., 2007; Talanian et al., 2007; Talanian et al., 2010; Frandsen et al., 2022) found no pre- and post-physical intervention changes in CD36 values. Concerning the variation of FAT/CD36 following diet and exercise intervention, Arkinstall et al. (2004) showed that FAT/CD36 increased after acute consumption of low carbohydrate (L-CHO) and short-term exercise. Glucose ingestion during MICT inhibits CD36 expression (Civitarese et al., 2005). FAT/CD36 is greater in high fat (H-FAT) during the recovery period after MICT than in high carbohydrate (H-CHO) (Hammond et al., 2016). Neither exercise nor CHO availability impacted CD36 mRNA expression (Hearris et al., 2019). Riis et al. (2019) showed no difference in FAT/CD36 in low carbohydrate (L-CHO) compared to H-CHO during exercise. In terms of the association between fat oxidation and FAT/CD36 expression during physical effort, Maunder et al. (2022) demonstrated a correlation between FAT/CD36 expression and peak whole-body fat oxidation during exercise. The genotypes TT of CD36 SNPs rs1527479 and GG of CD36 rs1984112 were associated with higher rates of whole-body fat oxidation (Jayewardene et al., 2016). Six weeks of HIIT (3 days/week) increased fat oxidation and FAT/CD36 (Perry et al., 2008). Physical training increased fat oxidation and FAT/CD36 expression (Tunstall et al., 2002; Schenk and Horowitz, 2006). The AA genotype of rs1761667 in the CD36 gene was associated with greater total fat intake (Fujii et al., 2019). In insulin-resistant participants, training increased adipose tissue vasculature and decreased FAT/CD36 expression in abdominal subcutaneous adipose tissue (Honkala et al., 2020). In individuals with type 2 diabetes, young people, and trained subjects, no significant differences were found in the change of FAT/CD36 protein expression in muscle after exercise between groups (Bruce et al., 2003). In response to 90 min of exercise at 60% VO_2_ peak, FAT/CD36 protein expression in muscle was higher in women than in men (Kiens et al., 2004).

Regarding the strength and reliability of our findings in this systematic review, we found: (i) Improvement in FAT/CD36 during or after exercise training is considered moderate evidence (1 high quality, 4 medium quality, and 1 low-quality study, respectively). (ii) The absence of changes in FAT/CD36 before and after a physical effort is classified as moderate evidence (0 high-quality, 3 medium-quality, and 1 low-quality studies, respectively). (iii) A correlation between FAT/CD36 expression and peak whole-body fat oxidation during exercise is also considered moderate evidence (1 high-quality, 5 medium quality, and 1 low-quality studies, respectively). (iv) Finally, the level of evidence for the association between FAT/CD36 and dietary intervention was judged to be very limited, given the considerable methodological and statistical heterogeneity between the studies.

## 4 Discussion

This systematic review examined the level of FAT/CD36 expression during exercise training and its association with adipose tissue lipolysis, both during exercise training alone and in combination with dietary intervention. The main findings indicate that the majority of studies on the association between FAT/CD36 expression and physical effort reported increases in FAT/CD36 both during and after physical effort. A correlation between FAT/CD36 expression and peak whole-body fat oxidation during exercise was observed in all studies investigating the association between fat oxidation and FAT/CD36 expression during exercise training. Some studies showed an increase in FAT/CD36 expression after an acute L-CHO and H-FAT, while others found no significant difference.

### 4.1 Regulation of CD36 and fatty acids oxidation

Lipolysis of adipose tissue during exercise training depends on several parameters: (i) duration and intensity of physical effort, (ii) aerobic capacity and VO_2_ peak, (iii) metabolic capacity, and (iv) the content of proteins required for FA transport, (v) lipolytic enzymes and accessory proteins (Li et al., 2022). In addition, different muscle fiber types influence the ability to oxidize FAs during exercise (Muscella et al., 2020). Type 1 (I) muscle fibers experience a greater reduction in intramuscular triglycerides (IMTG) than type 2 (II) muscle fibers, as type 1 (I) fibers contain approximately twice as much IMTG as type 2 (II) fibers (van Loon et al., 2001; De Bock et al., 2005). Highly trained endurance athletes exhibit greater peak fat oxidation due to the presence of more type I muscle fibers, which express higher levels of adipose triglyceride lipase (ATGL), 3-hydroxyacyl-CoA dehydrogenase (HAD), perilipin-5 (PLIN5), hormone-sensitive lipase (HSL), and oxidative phosphorylation (OXPHOS) (Shaw et al., 2020). This ability to use large amounts of FAs may be due to mitochondrial adaptations of fat oxidation and the mitochondrial volume density of the muscles involved (Holloszy, 1967; Horowitz and Klein, 2000). Mitochondrial volume density, mitochondrial fat oxidation, VO_2_ peak, and MFO are higher in endurance athletes than in controls, suggesting that MFO and mitochondrial volume density are associated with endurance athletes and may have an impact on athletic performance (Dandanell et al., 2018). The same study reported a strong correlation between mitochondrial volume density and MFO, suggesting that MFO and mitochondrial volume density are associated with endurance training (Dandanell et al., 2018). Furthermore, it has been shown that elite endurance athletes have a significantly higher mitochondrial volume density than untrained subjects (Hoppeler et al., 1973; Hoppeler et al., 1985). A study by Hetlelid et al. showed that fat oxidation is three times higher in elite runners than in non-elite (Hetlelid et al., 2015). Fat oxidation was 17 times higher in an athlete than in an untrained individual during HIIT, while CHO oxidation did not change in either group (Aslankeser and Balcı, 2017). Male endurance athletes show increased levels of fat oxidation products, such as dicarboxylates and monohydroxy fatty acids, acylcarnitine, and ketone bodies, after running (Nieman et al., 2017). At the same time, there is a reduction in the amount of muscle glycogen (Wolfe et al., 1990). The glycolytic flux increases during physical training and the increased production of pyruvate leads to an excess of acetyl-CoA, which in turn is buffered by catalase (CAT) (Muscella et al., 2020). The CAT enzyme converts excess acetyl-CoA into acetylcarnitine, which reduces the inhibition of pyruvate dehydrogenase complex (PDH) activity by acetyl-CoA produced by glycolysis, as occurs during intense exercise (Holness and Sugden, 2003). This buffering of acetyl-CoA allows for a high rate of pyruvate oxidation while reducing free carnitine content and limiting the mitochondrial import of FAs and therefore FA oxidation (Lundsgaard et al., 2018). The inhibition of the PDH is thus lifted, increasing glucose oxidation and promoting ATP resynthesis (Harris et al., 2002). The conversion of FAs to fatty acyl-CoA esters by Acyl-CoA synthetase (ACS) establishes a gradient that facilitates their uptake into the mitochondria (Muscella et al., 2020). Several isoforms of ACS are present in skeletal muscle cells, each with distinct subcellular locations and varying affinities for FAs. Of these isoforms, the long-chain acyl-coenzyme A synthetase (ACSL 1) plays a crucial role in fat oxidation in skeletal muscle during exercise (Lundsgaard et al., 2018). Several transport proteins facilitate the passage of FAs across the plasma membrane, such as FAs transport protein (FATP), FAs binding protein of the plasma membrane (FABPpm) and FAT/CD36 (Harasim et al., 2008). During exercise, there is a translocation of FAT/CD36 from intracellular reserves to the mitochondrial membrane in the muscle (Holloway et al., 2006; Monaco et al., 2015), in which it interacts with ACS to regulate the supply of fatty acyl-CoA to carnitine palmitoyl transferase 1 (CPT1) (Smith et al., 2011). Moreover, an increased expression of FAT/CD36 and total lipid oxidation was demonstrated after 9 days of endurance exercise (Tunstall et al., 2002). This suggests that FAT/CD36 is a critical factor in the regulation of fatty acid oxidation during exercise training (Muscella et al., 2020). Translocation of this protein from intracellular stores to the mitochondrial membrane allows for more efficient uptake and oxidation of FAs, thereby improving the metabolic capacity of muscle to meet the increased energy requirements of physical activity.

FAT/CD36 is an 88 kDa multifunctional glycoprotein and the main transporter of LCFAs from adipose tissue to the heart, skeletal muscle cells, and mitochondria (Ibrahimi et al., 1999; Bonen et al., 2007; Yanai et al., 2007). Fifteen exons encode FAT/CD36 and are composed of a single chain of 472 amino acids (Fernández-Ruiz et al., 1993; Rać et al., 2007). FAT/CD36 and CPT1 proteins coimmunoprecipitate in skeletal muscle and are both present in the outer mitochondrial membrane after endurance training (Schenk and Horowitz, 2006). FAT/CD36 is positioned on the outer mitochondrial membrane and contributes to the activation, regulation, and mitochondrial transport of FAs (Zeng et al., 2022). FAT/CD36 is situated on the sarcolemmal membrane and in the endosome, however, exercise can cause its reversible translocation from the sarcolemma to the plasma and mitochondrial membrane to facilitate transmembrane trafficking of FAs (Smith et al., 2012; Jordy and Kiens, 2014; Ferreira, 2018; Lundsgaard et al., 2018). During exercise training and muscle contraction under conditions of acute high-fat feeding, FAT/CD36 is transported from intracellular sites to the plasma and mitochondrial membrane by several molecules [including insulin, Ca^2+^, AMP-5-activated protein kinase (AMPK), extracellular signal-regulated kinase (ERK) and protein kinase C (PKC) (Arkinstall et al., 2004; Samovski et al., 2015; Ramos-Jiménez et al., 2022)] and interacts with caveolins in the sarcolemma under resting conditions (Momken et al., 2017). There is a strong relationship between AMPK activation, translocation of FAT/CD36 to the plasma membrane, and FA absorption and oxidation (Samovski et al., 2015). Additionally, in mouse muscle, leptin activates AMPK and induces translocation of FAT/CD36 into the plasma membrane, resulting in increased FAs uptake and oxidation (Momken et al., 2017). High-fat diet (Arkinstall et al., 2004), availability of LCFAs in tissues (Yun et al., 2020; Glatz et al., 2022), physical training (Tunstall et al., 2002; Frandsen et al., 2022) and muscle contraction (Rać et al., 2007; Yun et al., 2020Yun et al., 2020) stimulate FAT/CD36 expression, its insertion into the plasma membrane and its translocation to the cell cytosol. According to Jeppesen et al. (2011), FAT/CD36 protein content increases in the plasma membrane and decreases in the intracellular membranes during muscle contraction. FAT/CD36 plays a crucial role in the homeostasis and transport of LCFAs from the interstitial fluid to the cells for oxidation and ATP production (Karunakaran et al., 2021). The transport and oxidation of LCFAs in skeletal muscle are regulated by the expression, synthesis, and translocation of FAT/CD36 proteins (Glatz et al., 2022). In addition, activation of Peroxisome Proliferator-Activated Receptors (PPAR) transcription factors (the preferred ligand of FAs) by FAs is associated with increased expression and synthesis of FAT/CD36 (Glatz and Luiken, 2018). Through this regulation, FAT/CD36 supports the majority of energy requirements from lipid sources, particularly in metabolically active tissues such as skeletal muscle (Ramos-Jiménez et al., 2022). Finally, CD36 can undergo various post-translational modifications such as glycosylation, ubiquitination, palmitoylation, and acetylation, which can potentially impact FAT/CD36 processing and have regulatory implications (Su and Abumrad, 2009).

### 4.2 Effect of exercise training and nutrients on FAT/CD36

#### 4.2.1 Exercise training

Several studies have highlighted the role of FAT/CD36 in the absorption of FAs during physical exercise (Su and Abumrad, 2009). Studies involving CD36 in knock-out (KO) mice undergoing exercise (McFarlan et al., 2012), as well as in studies of *ex vivo* contracting muscle from transgenic mice overexpressing FAT/CD36 (Ibrahimi et al., 1999) demonstrate a critical role of FAT/CD36 protein levels in the regulation of fat oxidation and are supported by findings by Manio et al. (2017) that FAT/CD36 is crucial for the improvement of endurance performance after exercise training in mice. A deficiency of FAT/CD36 in humans decreases aerobic exercise capacity due to reduced FAs uptake by muscles (Yanai et al., 2007; Hames et al., 2014). FAT/CD36 plays an important role in the lipolysis of adipose tissue and the transport of FAs to the mitochondria for oxidation during exercise (Muscella et al., 2020; Glatz et al., 2022). The high levels of FAT/CD36 that occur during exercise increase lipid oxidation (Ferreira, 2018). Metabolic adaptation following chronic aerobic training increases oxidative capacity (Mahoney et al., 2005; Coffey and Hawley, 2007), with a correlation between FAT/CD36 abundance and an MFO (Maunder et al., 2022). Increases in FAT/CD36 and Plasma Membrane Fatty Acid-Binding Protein (FABPpm) of human skeletal muscle occurs after endurance cycling exercise at 60% VO_2_ peak (Bradley et al., 2012). A study by Yanai et al. (2007) observed a lower ventilatory threshold (VT) in participants with FAT/CD36 deficiency after 15 min of progressive exercise testing, with a correlation between VT and decreases in plasma FA (Yanai et al., 2007). A progressive increase in mitochondrial FAT/CD36 protein content (30%–60%) and LCFA transport and oxidation in skeletal muscle was measured during acute endurance exercise (120 min at 60% of VO_2_ peak) (Holloway et al., 2006). An increase in muscle FAT/CD36 content and mitochondrial respiratory capacity occurs after 8–16 weeks of endurance exercise (3 days per week, 20–40 min, 67%–80% of maximal HR) (Warren et al., 2020).

The impact of training intensity on fat oxidation and transport proteins can depend on several factors, including the type of exercise, duration, and frequency of training, and the individual’s fitness level. A correlation between greater HIIT duration or volume and a higher increase in fat oxidation has been demonstrated (Astorino et al., 2013). A single bout of high-intensity exercise in males leads to phosphorylation sites of 562 proteins (Hoffman et al., 2015). High-intensity training increases FA transport protein content in skeletal muscle (Talanian et al., 2010). Untrained individuals have an increased capacity for fat oxidation and carbohydrate metabolism in skeletal muscle after 18 h of HIIT program, with increases in the content and activity of the following oxidative and glycolytic proteins: β-hydroxyacyl-CoA dehydrogenase (β-HAD) was the most abundant protein (29%), followed by aspartate-amino transferase (AST) and citrate synthase (CS) (26%), PDH (21%), Cytochrome C Oxidase IV (Cox-IV), FAT/CD36, Fatty acid binding protein 4 (FABP4), glucose transporter protein (GLUT4, monocarboxylate transporter protein (MCT1 and MCT4) (14%–30%) (Perry et al., 2008). Mitochondrial content is improved after only 6-7 sessions of HIIT, which is associated with a higher fat oxidation capacity with HIIT (MacInnis and Gibala, 2017). In contrast, no increase in fat oxidation was observed after the initial HIIT phase in active and inactive women (Talanian et al., 2010; Astorino et al., 2013). These results suggest that intense exercise training may not lead to further improvements in fat oxidation above a certain threshold, indicating that there is a limit to the improvement in fat oxidation under various conditions. A sufficient intake of fatty acids and energy substrates is essential for fat oxidation, which could be limited by low-fat diets.

#### 4.2.2 Exercise training and diet interactions

Several studies demonstrated significant effects of CHO restriction in increasing adaptive responses to endurance training (Hansen et al., 2005; Cochran et al., 2010; Hulston et al., 2010; Bartlett et al., 2013; Lane et al., 2015). Reduced CHO availability before (Psilander et al., 2013), during (Akerstrom et al., 2006) and after (Pilegaard et al., 2005) training sessions enhances cellular signaling pathways and gene expression. In addition, markers of mitochondrial biogenesis (Hansen et al., 2005; Yeo et al., 2008a; Morton et al., 2009), whole body (Yeo et al., 2008a) and intramuscular lipid metabolism (Hulston et al., 2010) are increased by the reduced availability of CHO during short periods of endurance training, which was associated with improvements in exercise capacity and performance (Hansen et al., 2005; Marquet et al., 2016). Endurance training while fasting improves basal muscle fat transport and oxidative capacity, as well as FAT/CD36 and FABP protein content (De Bock et al., 2008; Van Proeyen et al., 2010). Dietary interventions such as L-CHO increase FAT/CD36, β-HAD, hormone-sensitive lipase (HSL), and uncoupling binding protein-3 (UCP3) (Arkinstall et al., 2004), while an acute fat diet increases gene expression of FATP, FAT/CD36, and β-HAD in skeletal muscles (Cameron-Smith et al., 2003). A high-fat diet after exercise does not increase mRNA expression of genes associated with regulatory roles in mitochondrial biogenesis, although it does increase the expression of lipid genes (Hammond et al., 2016). Three days of a hypercaloric and high-fat diet increases intramuscular triacylglycerol and FAT/CD36 mRNA levels (Jordy et al., 2014). Resting intramuscular triglyceride stores, CPT1, AMPK, hormone-sensitive lipase, and FAT/CD36 increases after 5–15 days of a high-fat diet (Goedec et al., 1999; Cameron-Smith et al., 2003; Yeo et al., 2008b). Decreases in CHO availability or a high-fat diet for five consecutive days reduce PDH activity and the ability to oxidize CHO (Stellingwerff et al., 2006), and lead to reduced athletic performance (Yeo et al., 2008a; Hulston et al., 2010). Post-exercise expression of FAT/CD36 was greater in H-FAT than in H-CHO, as reported in many studies of diet-exercise interventions (Cameron-Smith et al., 2003; Bartlett et al., 2013; Lane et al., 2015; Hammond et al., 2016).

Several studies have investigated sex differences in fat oxidation and FAT/CD36 expression during exercise training. For example, Kiens et al. (2004) reported that FAT/CD36 protein expression in skeletal muscle is higher in women than in men following exercise, regardless of training status. This sex difference in FAT/CD36 expression and fat oxidation can be explained by the differential specific content of each muscle fiber type (Ramos-Jiménez et al., 2022). On the contrary, there are no differences in fat oxidation after 2 weeks of sprint interval training (SIT) between men and women (Astorino et al., 2011), with the acute signaling response of genes involved in mitochondrial biogenesis due to SIT being mostly similar in males and females (Welle et al., 2008; Lundsgaard and Kiens, 2014; Skelly et al., 2017). Skeletal muscle in women tends to have more type I muscle fibers than in men (Cameron-Smith et al., 2003; Jordy et al., 2014), while men have a higher proportion of type IIA or both IIA and IIX muscle fibers than women (Jeon et al., 2019; Maher et al., 2010). The higher content of β-oxidation enzymes and LCFA flux in women contributes to sex differences in FAT/CD36 protein content in skeletal muscle (Yeo et al., 2008b; Jordy et al., 2014). No sex differences were observed in the relative contribution of CHO and fat to oxidative metabolism in the leg at rest and during submaximal exercise (90 min duration and 58% of VO_2_ peak) (Roepstorff et al., 2002). However, further research is needed to fully understand these differences and their implications for physical performance and health.

### 4.3 Limitations

The aim of this investigation was to assess the link between FAT/CD36 and lipolysis of adipose tissue in response to training. However, our systematic review included 21 studies with a relatively small total sample size (*n* = 859), hence our findings have to be viewed with some degree of caution. Furthermore, the issue of sex hormone-derived differences in fat oxidation is not addressed herein as many of the existing studies have ignored this issue (Oosthuyse et al., 2023). More research is needed on the topic of FAT/CD36 to facilitate the overcoming of these issues.

## 5 Conclusion

In conclusion, our study confirms the association between FAT/CD36 and lipolysis of adipose tissue during physical training and highlights a correlation between peak fat oxidation and FAT/CD36 expression. Furthermore, an increase in FAT/CD36 expression was observed after an acute phase of L-CHO and H-FAT. In contrast, no significant differences were observed under these conditions in other investigations. More specifically, and in terms of comparing the association between adipose tissue lipolysis and FAT/CD36 expression during physical training in athletes and non-athletes, our study found no convincing results. Finally, our study revealed contradictory results concerning FAT/CD36 expression during a dietary intervention combined with physical exercise.

## Figures and Tables

**Table:** 

**β-HAD**	β-Hydroxyacyl-CoA Dehydrogenase
**ACS**	Acyl-CoA Synthetase
**ACSL**	Long-chain acyl-coenzyme A Synthetase
**AMPK**	5-AMP-activated Protein Kinase
**AST**	Aspartate-amino Transferase
**ATGL**	Adipose Triglyceride Lipase
**ATP**	Adenosine Triphosphate
**CAT**	Catalase
**CD36**	Cluster of Differentiation 36
**CHO**	Carbohydrate
**Cox IV**	Cytochrome C Oxidase IV
**CPT1**	Carnitine Palmitoyl Transferase 1
**CS**	Citrate Synthase
**ERK**	Extracellular signal-Regulated Kinase
**FABP 4**	Fatty Acid-Binding Protein 4
**FABPpm**	Plasma Membrane Fatty Acid-Binding Protein
**FAs**	Fatty acids
**FAT/CD36**	Fatty Acid Translocase
**FATP**	FAs Transport Protein
**GLUT4**	Glucose Transporter Protein
**HAD**	3-Hydroxyacyl-CoA Dehydrogenase
**H-CHO**	High carbohydrate
**H-FAT**	High Fat
**HIIT**	High Intensity Intermittent Training
**HRmax**	Maximal Heart rate
**HSL**	Hormone-Sensitive Lipase
**IMTG**	Intramuscular Triglycerides
**LCFAs**	Long-chain Fatty acids
**L-CHO**	Low Carbohydrate
**MCT**	Monocarboxylate Transporter Protein
**MFO**	Maximal Fat Oxidation
**MICT**	Moderate Intensity Continuous Training
**OXPHOS**	Oxidative Phosphorylation
**PDH**	Pyruvate Dehydrogenase Complex
**PKC**	Protein Kinase C
**PLIN5**	Perilipin-5
**PPAR**	Peroxisome Proliferator-Activated Receptors
**UCP3**	Uncoupling Binding Protein-3
**VO2 peak**	Peak Oxygen Consumption

## Data Availability

The original contributions presented in the study are included in the article/Supplementary Material, further inquiries can be directed to the corresponding authors.
